# Subwavelength-scale off-axis optical nanomanipulation within Gaussian-beam traps

**DOI:** 10.1515/nanoph-2024-0527

**Published:** 2025-01-24

**Authors:** Lei-Ming Zhou, Wan Sun, Zong-Qiang Tao, Ning-Jun Xiong, Chan Huang, Xiao-Yun Jiang, Yu-Xuan Ren, Yuanjie Yang, Yu-Zhi Shi, Ji-Gang Hu, Qiwen Zhan

**Affiliations:** Department of Optical Engineering, School of Physics, 558979 Hefei University of Technology, Hefei, Anhui 230601, China; Institute for Translational Brain Research, MOE Frontiers Center for Brain Science, Fudan University, Shanghai, 200032, China; Department of Neurology, Jinshan Hospital, Fudan University, Shanghai, 201508, China; School of Physics, University of Electronic Science and Technology of China, Chengdu 611731, China; Institute of Precision Optical Engineering, School of Physics Science and Engineering, Tongji University, Shanghai 200092, China; School of Optical-Electrical and Computer Engineering, University of Shanghai for Science and Technology, Shanghai 200093, China

**Keywords:** optical manipulation, off-axis trapping, transverse scattering force, multiple potential-wells, particle sorting

## Abstract

It is generally recognized that there is only a single optical potential-well near the focus in optical traps with a focused Gaussian beam. In this work, we show that this classic Gaussian-beam optical trap has additional optical potential-wells for optical manipulation at the subwavelength scale in the off-focus transverse plane. The additional optical potential-wells are formed by the synergy of both the gradient trapping force and the transverse scattering force, though in previous studies the scattering force usually has adverse effect such as reducing trapping stability. These potential-wells work for not only the metallic particles, but also the high refractive-index dielectric particles. By engineering the contribution of the gradient force and scattering force through the particle size, the particle material and the position of the manipulation transverse plane, the force field and trapping potential-well can be tailored to trap/manipulate nanoparticles at different off-axis distance at the subwavelength scale. Our work provides new insight into optical tweezers and promises applications in optical nanomanipulation, nanoparticle sorting/separation, particle patterning and micro-fabrication on substrates.

## 1 Introduction

With one of the famous applications known as optical tweezers [1], [2], [3], [4], optical manipulation is a kind of technique that uses the mechanical effect of light to manipulate objects. Since its invention, it has found applications in many fields such as biology [5], [6], [7], [8], [9], atomic physics [10], [11], [12] and colloid science [13], [14], [15], [16], [17]. Additional to the gradient and scattering forces, there are also the lateral force [18], [19], [20], pulling force [21], [22] and micro-fluidic force [23], [24]. All these forces make the optical manipulation show great potential for particle sorting and biological cell screening [25], [26], [27], [28]. Recently, with cooling of the trapped particle in vacuum [29], it has aroused interest in the investigation of macro-quantum state [30], [31], [32], [33], nonequilibrium thermodynamics [34], [35] and high-precision measurement applications [36], [37], [38].

Typically, particles are trapped at the focal point of the focused Gaussian laser beam through optical gradient force [39]. However, off-axis trapping is needed in some scenarios including both practical applications and physics investigations. For the realization of orbital motion of particles, off-axis trapping is the fundamental step to build the orbits of trapped particles [40], [41], [42]. For the optical trapping/manipulation and screening of biological cells, strong optical fields tend to damage the cells [8], [43]; so it will be benefited by the off-axis trapping with low light intensity [44]. For optical trapping of nonlinear materials by femtosecond laser beam, there are various off-axis trapping phenomena to investigate [45], [46], [47]. In these researches, the transverse scattering force plays a pivotal role.

In this work, we started from the investigation of the transverse scattering force in focused Gaussian beams and reported an off-axis optical manipulation scheme utilizing the transverse scattering force. It is found that in strongly focused Gaussian beams, there are off-axis potential-wells on the non-focal transverse planes at the subwavelength scale. These potential-wells can be used for off-axis trapping of both metallic particles and high refractive-index dielectric particles. This optical manipulation is achieved by the synergy of gradient trapping forces and transverse scattering forces. Because of the different characteristics of the gradient force and scattering force, the off-axis trapping location of particles depends on the particle sizes, the particle materials and the trapping beams. Based on this we also demonstrated nanoparticle sorting/separation at the subwavelength scale. Though few works have paid attention to the optical manipulation utilizing the transverse scattering force, we have noticed the previous related works for micrometer-size metallic particles [48], [49], [50]. Off-axis trapping of gold particles with distance of several micrometers have been observed and interpreted by the transverse scattering forces [48], [49], [50]. However, quantitive study of the transverse scattering for the nanoparticles for the first time here shows the characteristics of this system in the subwavelength scale. It’s found that off-axis trapping is not only possible for metallic particles with strong scattering force, but also possible for dielectric particles with high refractive index. This can put forward the optical nanomanipulation (i.e., optical manipulation of nanoparticles in the subwavelength scale) and can provide a method of particle separation/sorting at the subwavelength scale [51], [52], [53], [54], [55], [56]. It can also provide particle patterning and micro-fabrication techniques on the substrate [57], [58].

## 2 Results and discussions

### 2.1 Scheme and principles

**Figure 1:**
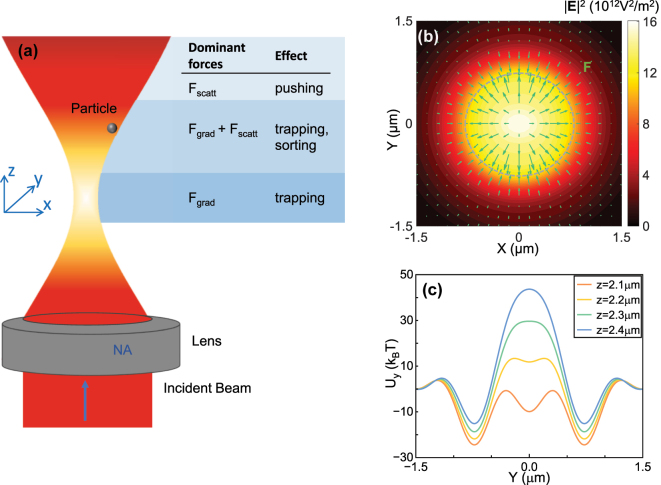
Off-axis optical manipulation at off-focus location utilizing the transverse scattering force. (a) Illustration of the setup for off-focus optical manipulation in a focused Gaussian beam. (b) Field intensity ${\left|E\right|}^{2}$ and force field **F** for a gold nanoparticle with radius *a* = 80 nm on the *xy*-plane when *z* = 2.3 μm. The gray dashed line denotes the locations where the value of the force in the radial direction is equal to zero. (c) Potential *U*_*y*_ along the *y*-direction for the gold nanoparticle when *z* = 2.1 μm, 2.2 μm, 2.3 μm, and 2.4 μm. The beam is an *x*-direction linearly polarized Gaussian beam propagating in the *z*-direction in water; NA = 0.9; light power *P* = 100 mW and vacuum wavelength *λ*_0_ = 1,064 nm.

The illustration of the trapping scheme is shown in Figure 1(a). An incident beam is focused by a lens to manipulate the particle, which is the typical setup of optical tweezers. Since we consider the transverse trapping, the stability of trapping along *z*-direction has been discussed in the Supplementary Material (SM) only and can be realized by a counter-propagating beam or a substrate/cover-plate in the *xy*-plane. The lens usually has a high numerical aperture (NA). The tightly focused beam was modeled by vector diffraction theory and calculated with Debye-Wolf integral [32], [59]. Since the accurate calculation of the focused beam field is the foundation of the analysis of optical forces, we have analyzed the error of simulated focused beams in the Supplementary Material. The result shows the correctness of the calculated electromagnetic field used in our scheme. The forces exerted on particles were then calculated with T-matrix method [32]. As a typical result for a gold nanoparticle trapping in water by a Gaussian beam, it can be seen that the force field in the transverse plane has zero values in the radial direction at off-axis locations (denoted by the grey dashed line Figure 1(b)) and the Gaussian beam can show off-axis potential-wells [Figure 1(c)].

### 2.2 Focused beam field at off-focus location and off-axis trapping

**Figure 2:**
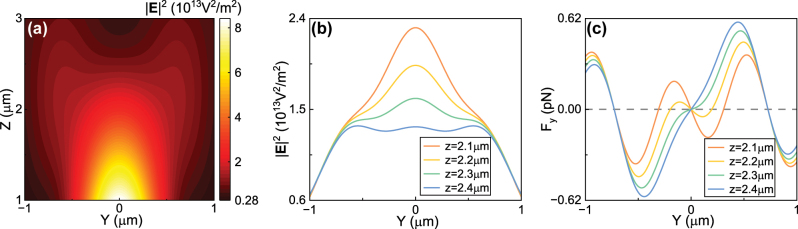
Field distribution of a focused Gaussian beam at the off-focus location and the off-axis optical trapping in water. (a) Intensity field ${\left|E\right|}^{2}$ on the *yz*-plane. (b) Electric field distribution along *y*-axis when *z* = 2.1, 2.2, 2.3, and 2.4 μm. (c) The total optical force *F*_*y*_ along *y*-axis for a gold nanoparticle with radius *a* = 80 nm. Other parameters are the same as those in Figure 1.

We have investigated the trapping performance of gold nanoparticles in water with our scheme of off-axis trapping. A Gaussian beam with *x*-direction polarization and a wavelength of 1,064 nm has been focused to irradiate the gold nanoparticles vertically as shown in Figure 1 (lens with NA = 0.9, incident light power *P* = 100 mW, nanoparticle radius *a* = 80 nm and permittivity *ɛ*_*p*_ = −47.21 + 1.436*i* for gold material [60]). The field intensity ${\left|E\right|}^{2}$ of the focused beam has been shown in Figure 2(a) for the longitudinal *yz*-plane with *x* = 0 μm and Figure 1(b) for the transverse plane with *z* = 2.3 μm. In Figure 1(b), we also showed the transverse force field. It can be seen that the radial component of the force has zeros at the off-axis locations. We also showed the intensity and the force *F*_*y*_ along *y*-axis on different transverse planes in Figure 2(b) and (c), respectively. It is clearly that when *z* = 2.3 μm, the light intensity has a single peak profile along the *y*-axis, but the *F*_*y*_ has zeros values at the off-axis location near *y* = 0.7 μm. The nanoparticle can thus be trapped at the off-axis locations on an off-focus transverse plane.

**Figure 3:**
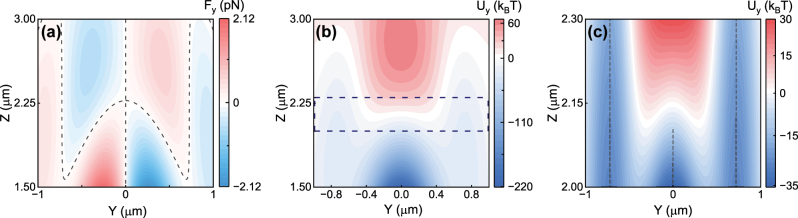
Numerically calculated forces and potential-wells of a gold nanoparticle with radius *a* = 80 nm for off-axis trapping in a focused Gaussian beam. (a) Force *F*_*y*_ on the *yz*-plane. The black dashed curve denotes the *F*_*y*_ = 0 positions. (b) Potentials *U*_*y*_ with unit of *k*_*B*_ *T* (*T* = 25 °C) on the *yz*-plane within the range of 1.5 μm < *z* < 3 μm. The area circled by the dark-blue dashed rectangular box will be magnified in figure (c). (c) Potentials *U*_*y*_ on the *yz*-plane for the gold nanoparticle within the range of 2 μm < *z* < 2.3 μm. The black dashed lines mark the locations of the local minima of the potential *U*_*y*_. Other parameters are the same as those in Figure 1.

In order to investigate the off-axis trapping range along *z*-axis, we showed the result of the force distribution and potential-well of the gold nanoparticle on the *yz*-plane in Figure 3(a)–(c). It can be seen clearly that the gold nanoparticles can be trapped at off-axis location with 1.5 μm < *z* < 2.3 μm. We also noticed that there is off-axis trapping when *z* > 2.3 μm, which is the result of the abnormal light intensity distribution (we will discuss it in the Supplementary Material).

Revealing the mechanism of the off-axis trapping can help to tune and control this phenomenon. The off-axis trapping phenomenon is induced by the balance between the transverse gradient force and the transverse scattering force. Taking the *y*-axis as an example, the total transverse force can be approximately written as

(1) $$F_{y}\approx F_{y,\mathrm{grad}}+F_{y,\text{scat}},$$

where *F*_*y*,grad_ and *F*_*y*,scat_ are the gradient force and the scattering force respectively.

When the particle is small, we can take the Rayleigh approximation and formulate Eq. (1) as

(2) $$F_{y}=\frac{\text{Re}\left(\alpha \right)}{4}\nabla {\left|E\right|}^{2}+\frac{\sigma_{\text{ext}}n_{m}}{c}S_{y}.$$

Among Eq. (2), $\alpha =\alpha_{0}/\left(1-\frac{ik^{3}\alpha_{0}}{6\pi \varepsilon_{m}}\right)$ is the polarizability of the particle, in which $\alpha_{0}=4\pi \varepsilon_{m}a^{3}\frac{\varepsilon_{p}-\varepsilon_{m}}{\varepsilon_{p}+2\varepsilon_{m}}$ is the static polarizability; *ɛ*_*p*_ and *ɛ*_*m*_ are the permittivity of the particle and the surrounding medium; *a* is the particle radius; $\sigma_{\text{ext}}=k\text{Im}\left(\alpha \right)/\varepsilon_{m}$ is the extinction cross-section, *n*_*m*_ is the refractive index of the surrounding medium and *S*_*y*_ is the *y*-component of Poynting vector **S** of the focused beam.

**Figure 4:**
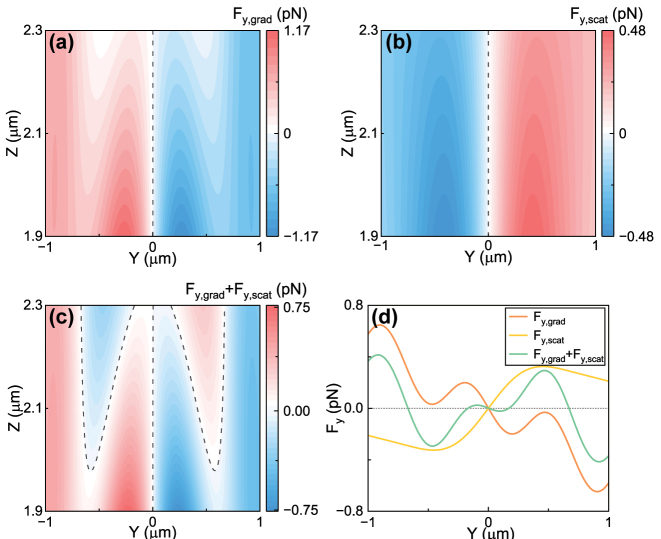
Numerically calculated forces of a gold nanoparticle with radius *a* = 80 nm for off-axis trapping in a focused Gaussian beam. (a) Force *F*_*y*,grad_ on the *yz*-plane. (b) Force *F*_*y*,scat_ on the *yz*-plane. (c) Force *F*_*y*,grad_ + *F*_*y*,scat_ on the *yz*-plane. (d) *F*_*y*_ along *y*-axis when *z* = 2.3 μm on the *yz*-plane. The black dashed curves in subfigures (a–c) mark the positions where the forces are zeros. Other parameters are the same as those in Figure 1.

The two terms on the right side of Eq. (2) are corresponding to the transverse gradient force and transverse scattering force, respectively. We defined them as $F_{y,\mathrm{grad}}=\frac{\text{Re}\left(\alpha \right)}{4}\nabla {\left|E\right|}^{2}$ and $F_{y,\text{scat}}=\frac{\sigma_{\text{ext}}n_{m}}{c}S_{y}$ . We calculated these two terms and showed the results in Figure 4(a) and (b), respectively. It can be seen that these two forces have opposite directions and the comparable magnitude.

The total transverse force *F*_*y*_ in Eq. (2) is then calculated and shown in Figure 4(c). The result suggests the off-axis equilibrium points as denoted by the dashed lines at the off-focus locations. It’s noted that there are stable and metastable equilibrium points. Only the most out lines and the central dashed line denote the stable trapping points. We have also showed the forces *F*_*y*,grad_, *F*_*y*,scat_ and *F*_*y*,grad_ + *F*_*y*,scat_ along *y*-axis when *z* = 2.3 μm in Figure 4(d). It’s clear that there are three trapping points on the *y*-axis.

### 2.3 Tunability of the off-axis trapping system

With the above theory, the off-axis trapping behaviors depend on both the gradient force and the scatting force. According to Eq. (2), we can tune the forces in various ways, including the NA of the lens, the location of the off-focus plane, the particle size and the particle materials. With different NA of lenses, both the light field and the energy flux have different distributions. Since tuning NA is not the convention in optical manipulation, we showed its result in the Supplementary Material. It is found that the off-axis trapping region of the gold nanoparticle is closer to the focal plane when the NA increases. Also, it is found that the range with off-axis trapping in the *z*-direction decreases when the NA increases.

**Figure 5:**
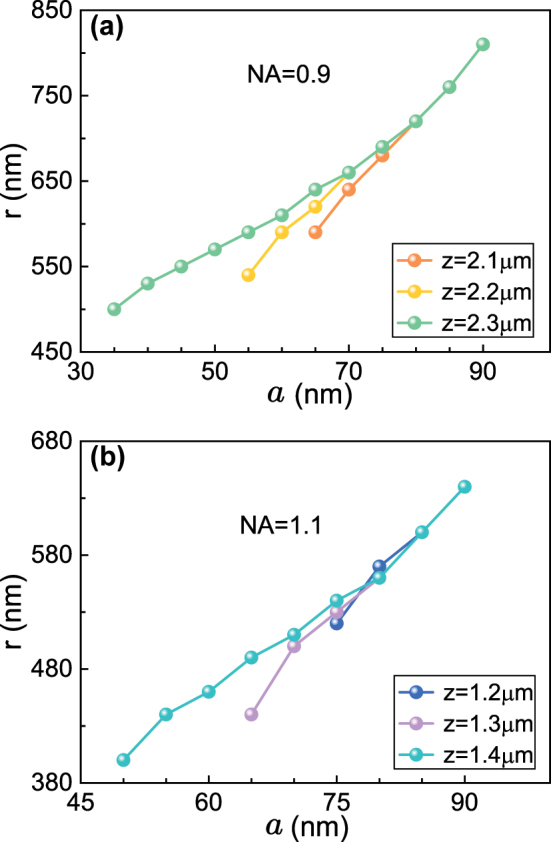
Numerically calculated off-axis trapping positions within a focused Gaussian beam with different particle radii and different numerical apertures of the objective lenses. (a) Off-axis trapping positions of gold nanoparticles with different radii for NA = 0.9 at *z* = 2.1, 2.2, and 2.3 μm. (b) Off-axis trapping positions with different particle radii for NA = 1.1 at *z* = 1.2, 1.3, and 1.4 μm. Other parameters are the same as those in Figure 1.

With different particle radii, the particles have different polarizability and can change the ratio between different force components. The gradient force follows an index law of *a* ^3^ and the scattering force is proportional to *a* ^6 ^[39]. With different particle radii, their proportions in the total force will change. This mechanism can be used to engineer the off-axis trapping locations and tailor the optical potential-wells. The off-axis trapping position on the *y*-axis generally increases with the increasing radii of the gold nanoparticle, and this has been shown in Figure 5 for different transverse planes and different numerical apertures of objective lenses. It’s also observed that there is no off-axis trapping phenomenon for the particles having very small radii.

### 2.4 Separation/sorting of metallic particles

**Figure 6:**
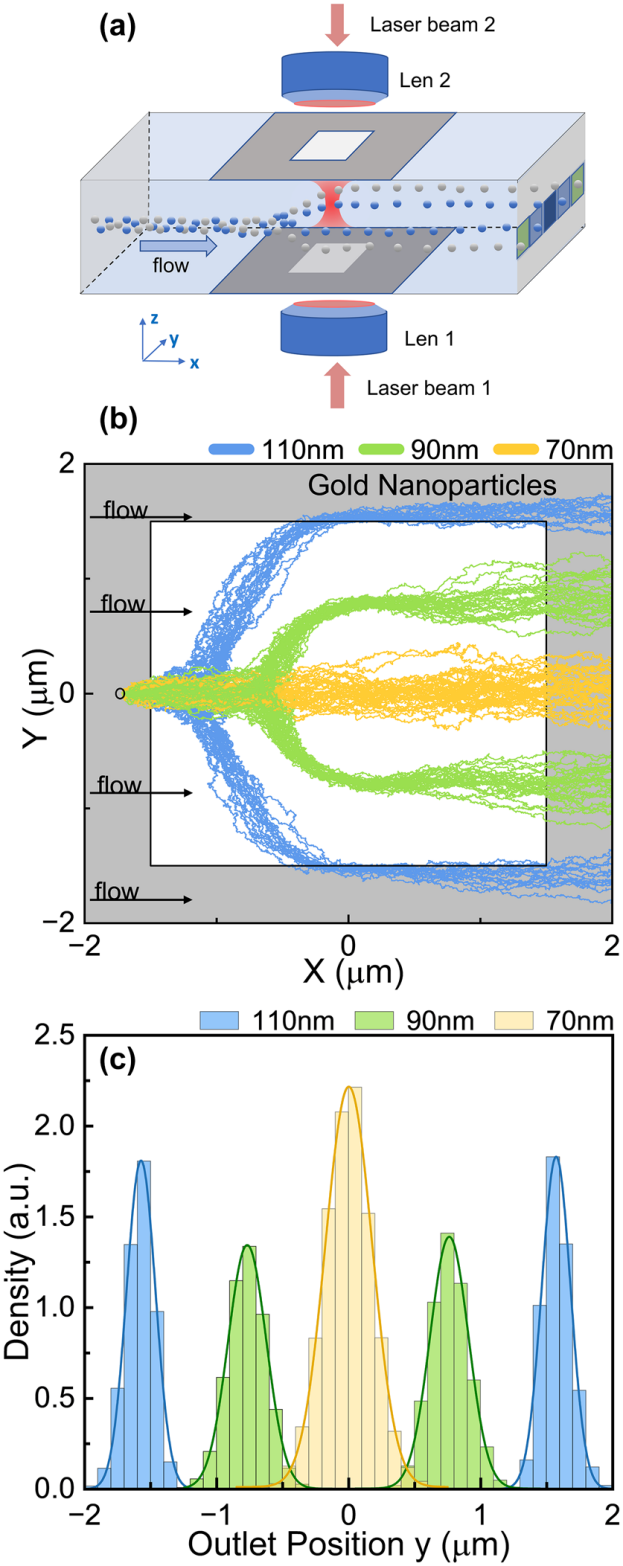
Demonstration of gold nanoparticle separation/sorting. (a) Illustration of the setup for particle sorting. (b) Simulated trajectories of gold nanoparticles with different radii *a* in the *xy*-plane when *z* = 2.2 μm. The light field is limited in the white square of side length 3 μm by masks. The velocity of water flow is 500 μm/s. (c) Particle distributions of different gold nanoparticles at the outlet (*x* = 2.0 μm). Other parameters are the same as those in Figure 1.

Taking the advantage of the tunability of this off-axis trapping system, we explored the possibility of sorting/separating nanoparticles. For particle separation, we first demonstrated the separation of gold nanoparticles of different sizes. We designed a very simple setup as shown in Figure 6(a), which consisted of a microfluidic channel along *x*-axis, focused beams along the *z*-axis and a mask having the light input window. The focused light beam can pass through the input window into the fluid without change of the channel. As an example, gold nanoparticles with radii 110 nm, 90 nm, and 70 nm have been released at the input point (−1.7, 0) µm on the transverse plane and into water flow with current velocity of 500 μm/s. The gold nanoparticles then undergo different trajectories driven by the resultant force consisted of the optical forces, fluidic drag forces, and Brownian forces [Figure 6(b)]. It can be seen that the particles with different size have got different *F*_*y*_ and thus different displacements along *y*-axis within the 3.0 μm × 3.0 μm light window (denoted by the white area).

To illustrate the sorting effect, we plotted the outlet distributions of gold nanoparticles in Figure 6(c). They are separated very well as shown. The force field of different particles during separation has also been shown in the Supplementary Material. It can be seen that the transverse scattering force in the off-focus plane plays important role in this sorting system within several micrometer range.

### 2.5 Separation/sorting of dielectric particles with high refractive index

**Figure 7:**
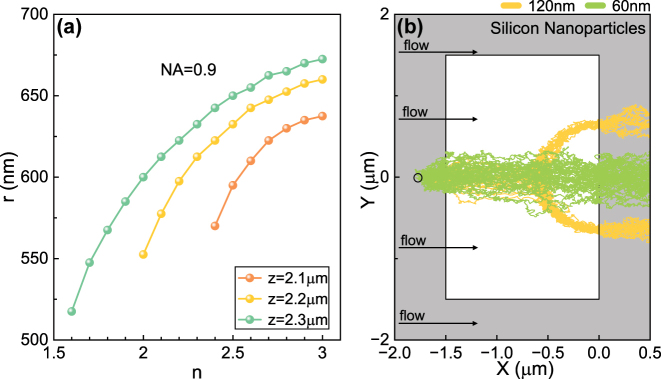
Numerically calculated off-axis trapping and sorting of dielectric particles with different refractive index in Gaussian beams. (a) Off-axis trapping positions of 120 nm-radius dielectric particles with different refractive indices for NA = 0.9 at *z* = 2.1, 2.2, and 2.3 μm (b) simulated trajectories of silicon nanoparticles with different radii *a* in the *xy*-plane when *z* = 2.2 μm. The light field is limited with the 1.5 μm × 3 μm light window denoted by the white area. The velocity of water flow is 150 μm/s. Other parameters are the same as those in Figure 1.

With different particle materials, the particles will also have different polarizability. The real part of the polarizability (i.e., $\text{Re}\left(\alpha \right)$ ) and extinction cross-section *σ*_ext_ will also change and thus engineer the trapping behaviors. We have investigated the off-axis trapping positions in the *y*-axis for dielectric particles of different materials (i.e., refractive index) in Figure 7. As shown in Figure 7(a), the off-axis trapping position of the dielectric particles in the off-focus plane changes as the refractive index of the particles increases. It’s also observed that there is no off-axis trapping phenomenon for the particles having low refractive index. Based on these results, we also demonstrated the sorting of silicon nanoparticles in Figure 7(b) based on particle size. The permittivity is *ɛ*_*p*_ = 12.63 + 0.0006*i* for silicon materials [61]. We also investigated the separation of particles of the same size but different materials in Figure S7 in the Supplementary Material. The results show the ability to sort these particles.

The key point of this particle separation scheme is to utilize the transverse scattering force of different particles (size or material). Different from the gradient force which always attracts all the particles to the field maxima, the transverse scattering force helps us to separate particles according to their size and material.

## 3 Discussions

As a discussion, it’s noted that the off-axis trapping does not happen in the Gaussian-beam optical tweezers for the most common particles, such as silica and polystyrene particles. This is because the transverse scattering force is smaller than the gradient force for these particles. However, for metallic particles and high refractive-index particles such as gold and silicon, they have a larger extinction cross-section to increase the scattering force, which makes the off-axis optical manipulation possible. Recently, high refractive index particles, such as silicon particles and nanodiamonds, have aroused interest with their applications in vacuum levitated-optomechanics and fundamental research [31], [32], [62]. Our proposal can also provide support for the particle preparations in these realms.

Secondly, the force in the longitude direction has also been discussed in the Supplementary Material. There are various methods to balance the particle in the target transverse plane, such as using gravity, static electric force and counter-direction optical force. We showed the result of using a counter-propagating beam in the Supplementary Material. The result shows that with a counter-propagating laser beam of off-set waist in the *z*-direction, the particle can be trapped in the off-focus transverse plane in Figure S9.

Also, it suggests that by replacing the linearly-polarized Gaussian beam with circularly-polarized beam, the potential-well can also be used to build rotation orbits. Especially, it will be able to realize the orbital rotation in Gaussian beams without auxiliary orbit, thus have the advantage of tuning ability in the applications of optical-driven micromachines and micro-robots [63], [64], [65], [66].

## 4 Conclusions

In conclusion, we have investigated the off-axis trapping of nanoparticles in the non-focus transverse planes of focused Gaussian beams. The transverse scattering force induced by the diverging of the focused light beam matters and contributes to building off-axis optical potential-wells in the transverse plane. This provides a mechanism for advanced optical micro/nano-manipulation. It’s found that off-axis trapping is not only possible for metallic particles with strong scattering force, but also possible for dielectric particles with high refractive index. The proposed system uses only Gaussian beams and high numerical aperture lenses. However, it is easily reconfigurable and can be readily used for particle separation/sorting. The result shows potential in nanoparticle sorting, particle patterning and micro-fabrication on substrates and so on. Our work can provide theoretical foundations in advanced optical manipulation and promises applications in optical-driven micromachines and micro/nano-rheology.

## 5 Methods

**Simulation of particle kinetics in the optofluidic system**. Nanoparticles in a fluid system are subjected to the optical forces, fluidic drag forces, and Brownian forces. The trajectories of these particles can be described by the Langevin equation

(3) $$m\frac{{\text{d}}^{2}}{\text{d}t^{2}}r\left(t\right)=F_{\text{opt}}-\gamma \left(\frac{\text{d}}{\text{d}t}r\left(t\right)-u\right)+\chi \left(t\right),$$

where *γ* = 6*πηa* is the friction coefficient of the particle in aqueous solution (*η* and *a* are the fluid viscosity and the radius of the spherical particle, respectively). The first term of Eq. (3) represents the inertial term of the nanoparticle. The second term **F**_opt_ is the optical force. The third term of Eq. (3) represents the fluidic drag force. The last term $\chi \left(t\right)=\sqrt{2\gamma k_{B}T}W\left(t\right)$ represents the Brownian forces. The $W\left(t\right)$ denotes the Gaussian white noise. The *k*_*B*_ and *T* are the Boltzmann constant and the absolute temperature, respectively.

To simulate the trajectories of particles, the Langevin equation can be solved numerically using its finite-difference form. However, it is essential to ensure that the time step Δ*t* is considerably smaller than the characteristic time scales (such as the momentum relaxation time *τ*_*m*_ = *m*/*γ*) so that the trajectories can be simulated correctly [39]. In our case, the sphere particle has a characteristic dimension *d* ≈ 200 nm and is moving at velocity ${\left|v\right|}_{\max}\approx 1\text{,}000\;\mu m/\text{s}$ through water with viscosity *η* ≈ 0.890 × 10^−3^ Pa s (when *T* = 25 °C) [67]. The density of the water is *ρ* = 1,000 kg/m^3^. Thus, the Reynolds number is Re = d*vρ*/*η* which is evaluated to be about 2.2 × 10^−4^. Under the condition of low Reynolds number $\left(\text{Re}\leq 100\right)$ , viscosity is predominant over inertia. Thus, we dropped the inertial term in Eq. (3) and solved the overdamped Langevin equation

(4) $$F_{\text{opt}}-\gamma \left(\frac{d}{dt}r\left(t\right)-u\right)+\chi \left(t\right)=0$$

to simulate the particle motion. The relevant characteristic time scale for Eq. (4) is *τ*_ot_ = *γ*/*κ*, where *κ* is the stiffnesses of the optical trap. In our simulation, *τ*_ot_ is about several milliseconds, while *τ*_*m*_ is about 50 ns. Solving the overdamped Langevin equation helps us to employ a relatively large time step and reduce the computation time.

## Supplementary Material

The Supplementary Material is attached as an independent file, including contents: Focused beam field under high numerical aperture lens; Anomalous electromagnetic field distribution on the off-focus plane; Off-axis trapping in different radial directions in a linearly polarized Gaussian beam; Off-axis trapping for different NA; The force field of different size particle during separation; Separation/sorting particles with different materials; 3D optical trapping using an additional counter-propagating beam.

## Supplementary Material

Supplementary Material Details
