# Supplementary material for: Subwavelength-scale off-axis optical nanomanipulation within Gaussian-beam traps
Source: Nanophotonics. 2025 Jan 24;14(2):219–28. doi: 10.1515/nanoph-2024-0527 (PMC11806509; doi:10.1515/nanoph-2024-0527)
Supplement: Supplementary file 1 — Supplementary Material Details [file j_nanoph-2024-0527_suppl_001.docx]

Supplementary Material to:

Subwavelength-scale off-axis optical nanomanipulation within Gaussian-beam traps

Lei-Ming Zhou^1^, Wan Sun^1^, Zong-Qiang Tao^1^, Ning-Jun Xiong^1^, Chan Huang^1^, Xiao-Yun Jiang^1^, Yu-Xuan Ren^2,3^, Yuanjie Yang^4^, Yu-Zhi Shi^5^, Ji-Gang Hu^1^^,*^, and Qiwen Zhan^6^

^1^Department of Optical Engineering, School of Physics, Hefei University of Technology, Hefei, Anhui 230601, China

^2^Institute for Translational Brain Research, MOE Frontiers Center for Brain Science, Fudan University, Shanghai, 200032, China

^3^Department of Neurology, Jinshan Hospital, Fudan University, Shanghai, 201508, China

^4^School of Physics, University of Electronic Science and Technology of China, Chengdu 611731, China

^5^Institute of Precision Optical Engineering, School of Physics Science and Engineering, Tongji University, Shanghai 200092, China

^6^School of Optical-Electrical and Computer Engineering, University of Shanghai for Science and Technology, Shanghai 200093, China

^*^Correspondence: J.-G. H. ([hujigang@hfut.edu.cn](mailto:hujigang@hfut.edu.cn)).

1. Focused beam field under high numerical aperture lens

The field distribution of the scattering force in focused beam depends on the numerical aperture (NA) of lens. Accurate calculation of the electromagnetic field distribution of the focused beam is foundation of the analysis of optical forces and optical potential-wells. Here, we used Debye integral (vector diffraction integral) to calculate the focused electromagnetic field [1]:

$\mathbf{E}_{\mathrm{debye}}\left( \rho,\varphi,z \right)=-\frac{ikfe^{-ikf}}{2\pi}\int_{0}^{\theta_{\max}} \int_{0}^{2\pi} \mathbf{E}_{\infty}(\theta,\phi)e^{ikz\cos\theta}e^{ik\rho\sin\theta cos(\phi-\varphi)}\sin\theta d\phi d\theta.$ (S1)

In Eq. (S1), $\mathbf{E}_{\infty}(\theta,\phi)$ is the field on the reference surface of the focusing lens.

As an example, here we first calculated the distribution of the electric field intensity $\left| \mathbf{E}_{\mathrm{debye}} \right|^{2}$ in the $yz$-plane with the Debye integral for a Gaussian beam focused by a lens of $\mathrm{NA}=0.9$, and showed the result in Fig. S1(a). The surrounding medium is water; the incident beam is $x$-direction linearly polarized with the wavelength of 1064 nm and power of 100 mW. Before calculating the optical force, we calculated the error $\left| \mathbf{E}_{\mathrm{error}} \right|^{2}$ of the result of Debye integral for the optical field and showed the error in Fig. S1(b). The error calculation has been finished by the finite element method (FEM). The total field of focused beam can be written as

$\mathbf{E}=\mathbf{E}_{\mathrm{debye}}+\mathbf{E}_{\mathrm{error}}.$ (S2)

If we set the $\mathbf{E}_{\mathrm{debye}}$ as a background field for an empty scattering problem of electromagnetic field, the scattering field can be regarded as the error. It can be seen that relative error $\left| \mathbf{E}_{\mathrm{debye}} \right|^{2}/\left| \mathbf{E}_{\mathrm{error}} \right|^{2}$ is very small and can be omitted in Fig. S1(c).


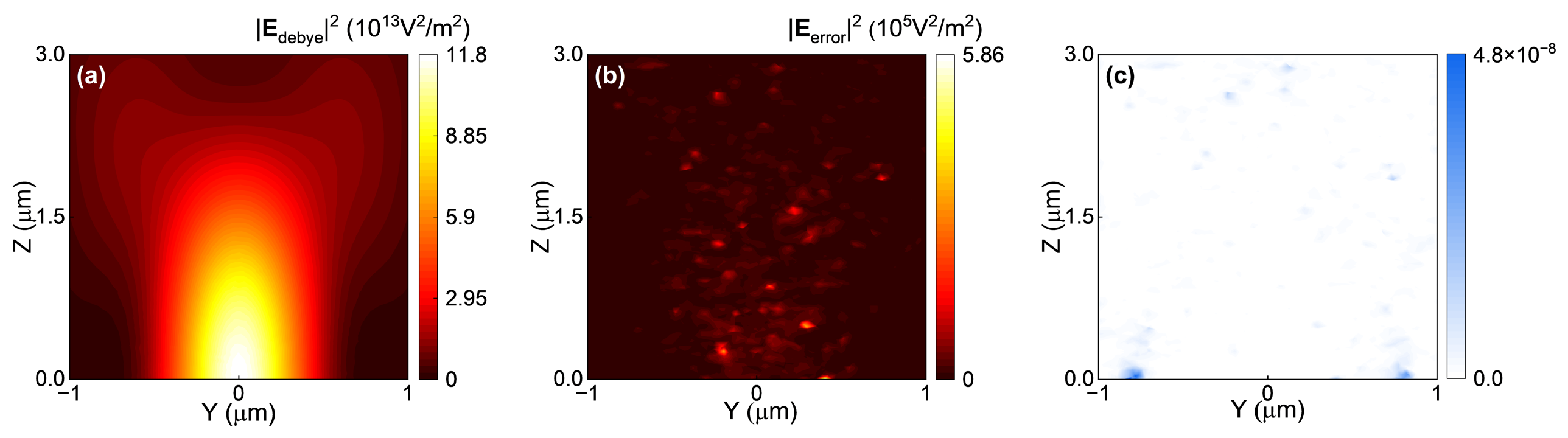


Fig. S1. (a) Distribution of field intensity $\left| \mathbf{E}_{\mathrm{debye}} \right|^{2}$ on the $yz$-plane. (b) The Error $\left| \mathbf{E}_{\mathrm{error}} \right|^{2}$ of Debye integral on the $yz$-plane. (c) Relative error $\left| \mathbf{E}_{\mathrm{debye}} \right|^{2}/\left| \mathbf{E}_{\mathrm{error}} \right|^{2}$ on the $yz$-plane. The beam was selected as an $x$-linearly polarized Gaussian beam with a wavelength of 1064 nm propagating in the $z$-direction in water; $\mathrm{NA}=0.9$; light power $P=100 \mathrm{mW}$.

1. Anomalous electromagnetic field distribution on the off-focus plane

We noticed that for $\mathrm{NA}=0.9$, the electric field distribution near the plane $z=2.5 \mu m$ is abnormal. It has a double peak as shown in Fig. S2. This field distribution can provide a double potential-well induced by the gradient force, which tends to attract particles to the region of highest light intensity. When the off-axis trapping in this situation happens, the gradient force always dominates, which is similar to the case of doughnut beams, so we won’t discuss it further.


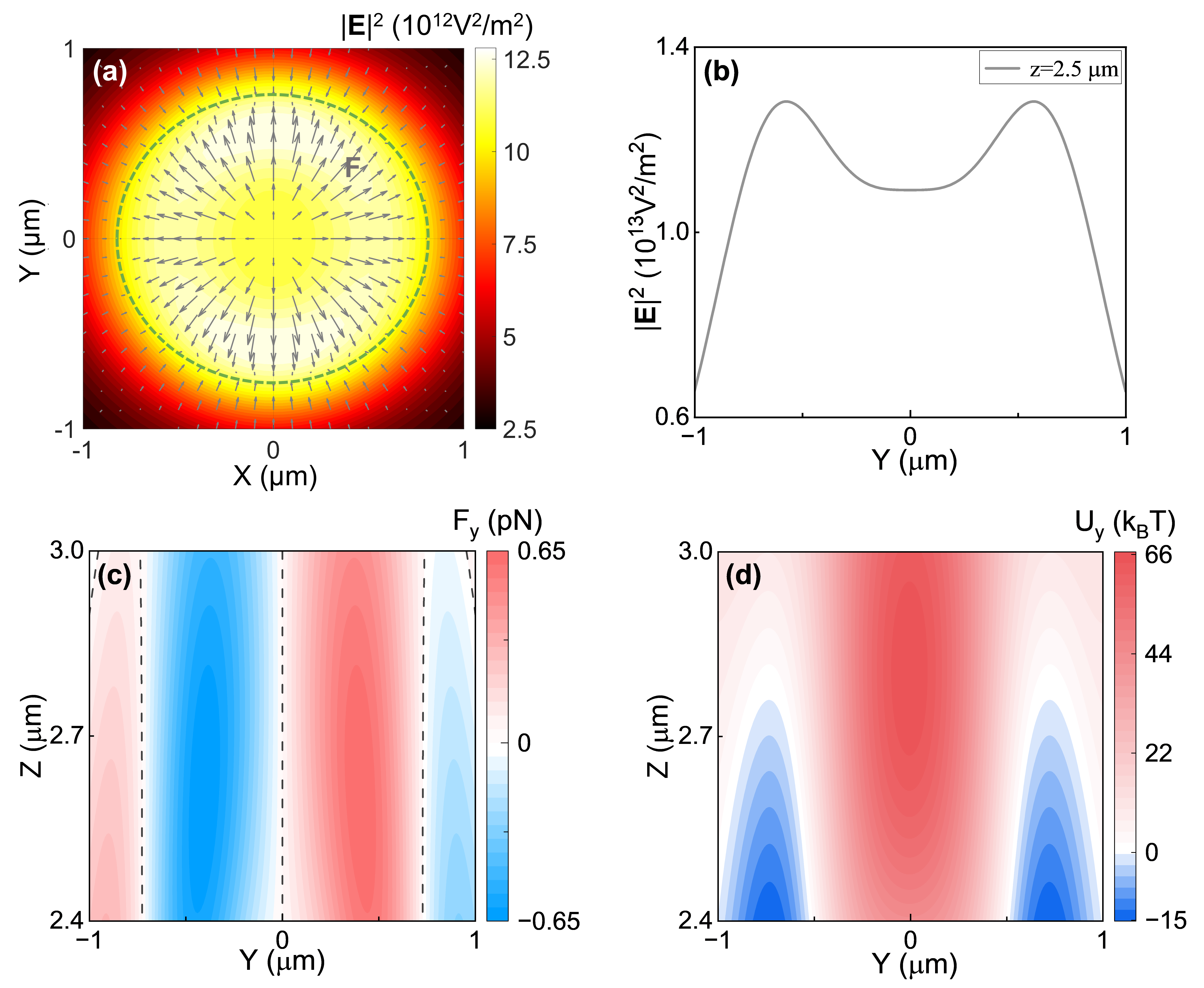


Fig. S2. Field distribution of a focused Gaussian beam at the off-focus location and off-axis trapping result of a gold particle with a radius of 80nm. (a) Electric field and force field distribution on the $xy$-plane when $z=2.5 \mu m$. The green dashed line plots the locations where the value of the force field is equal to zero. (b) Electric field distribution along $y$-axis when $z=2.5 \mu m$. (c) Force $F_{y}$ on the $yz$-plane for a gold particle within therange of $2.4 \mu m<z<3.0 \mu m$. The black dashed curve denotes the $F_{y}=0$ positions. (d) Potentials $U_{y}$ on the $yz$-plane for the gold particle within the range of $2.4 \mu m<z<3.0 \mu m$. Other parameters are the same as in Fig. S1.

1. Off-axis trapping in different radial directions in a linearly polarized Gaussian beam

It is known that the widths of the beam waists vary in different radial direction for a focused linearly polarized Gaussian beam. Thus, the off-axis trapping locations of particles in different radial direction are different. The focused linearly polarized beam has an elliptical intensity distribution approximately on its transverse plane as shown in Fig. 1b of the main text. As shown in Fig. S3(a), the intensity distributions in each radial direction are different even for the same transverse plane of $z=2.2 \mu m$ (three directions including the $x$-axis direction, the $y$-axis direction, and $y=x$ direction are shown here).

For a gold particle with a radius of 80 nm, the radial force $F_{r}$ and the potential $U_{r}$ in the three radial directions are shown in Fig. S3(b) and Fig. S3(c), respectively. The same case for the transverse plane of $z=2.3 \mu m$ is also shown in Fig. S3(d-f). As seen in Fig. S3(b) or Fig. S3(e), the difference between the off-axis trapping position in the $x$ -axis direction and that in the $y$ -axis direction is the most obvious (about 35 nm). In Fig.S3(c), the off-axis trapping position on the $x$-axis has a potential energy of about -24 $k_{B}T$ and the off-axis trapping position on the $y$-axis has a potential energy of about -33 $k_{B}T$. The potential depth differs by about 9 $k_{B}T$ at $z=2.2 \mu m$. And in Fig.S3(f), the potential depth along the two axes also differs by about 9$k_{B}T$ at $z=2.3 \mu m$.





Fig. S3. Field distributions along different radial directions on the off-focus plane and off-axis trapping results of a gold particle with a radius of 80nm. The three different radial directions plotted here are: the $x$-axis, the $y$-axis, and the $y=x$ direction. (a)-(c) ${|\mathbf{E}|}^{2}$, $F_{r}$ and $U_{r}$ in the three different radial directions for $z=2.2 \mu m$ plane. (d)-(f) ${|\mathbf{E}|}^{2}$, $F_{r}$ and $U_{r}$ in the three different radial directions for $z=2.3 \mu m$ plane. Other parameters are the same as in Fig. S1.

In order to show the trapping difference in each direction of the $xy$-plane clearly, we also plotted the $x$-direction force $F_{x}$ and $y$-direction force $F_{y}$ for the $xy$-plane at $z=2.2 \mu m$ in Fig. S4(a) and Fig. S4(b), respectively. In addition, Fig. S4(c) shows the optical potential in the radial direction of this $xy$-plane. As can be seen from the figure, the depth of the potential-well is deepest in the $y$-axis compared to the other radial directions. The particles thus tend to be trapped on the $y$-axis in the case of $x$-direction polarized incident beam here.





Fig. S4. Numerically calculated forces and potential-wells of a gold nanoparticle with a radius of $80\mathrm{nm}$ for off-axis trapping in a focused Gaussian beam. (a) The $x$-component of force $F_{x}$, (b) the $y$-component of force $F_{y}$ and (c) radial potential $U_{r}$ for the $xy$-plane at $z=2.2 \mu m$. Other parameters are the same as those in Fig. S1.

We have provided the calculation method for the potential in Fig. S3 and Fig. S4 in this paragraph. The potential $U_{r}$ is calculated using the following integral expression:

$U_{r}\left( r \right)=-\int_{0}^{r} F_{r}(r)dr$………………………………(S3)

Here, $F_{r}(r)$ represents the radial force at each position $r$ along the radial direction. This means that we choose the (x,y)=(0,0) as the point with zero energy (reference point of potential).

1. Off-axis trapping for different NA

With different NA of the lens, both the light field and the energy flux have different distributions. Consequently, the gradient force and scattering force have different contributions in the total force. We showed the force component $F_{y}$ in Fig. S5(a-c) and the corresponding trapping potential $U_{y}$ in Fig. S5(d-f) of a gold particle with radius $a=80 \mathrm{nm}$ on the $yz$-plane for numerical apertures of 1.0, 1.1, and 1.2, respectively. In Figs. S5(a-c), it is found that the off-axis trapping region of the gold particle is closer to the focal plane when the NA increases. Also, it is found in Figs. S5(d-f) that the range with off-axis trapping in the z-direction decreases when the NA increases.





Fig. S5. Numerically calculated forces and potential-wells for off-axis trapping in a single Gaussian beam with different NA. (a)-(c) Force $F_{y}$ on the $yz$-plane for a gold particle for NA of 1.0, 1.1 and 1.2, respectively. The black dashed curve denotes the $F_{y}=0$ positions. (d)-(f) Potentials $U_{y}$ on the $yz$-plane for the gold particle for NA of 1.0, 1.1 and 1.2, respectively. Other parameters are the same as in Fig. S1.

1. The force field of different size particle during separation

We have investigated the trajectory deviations from the x-axis for particles with different sizes. This can help to explore the relationship between these deviations and the spatial distributions of the forces on the particles. For this purpose, we have plotted the force field of the particles in the $xy$-plane in Fig. S6, which shows the resultant force of optical force and fluid force on the particle at rest (with a flow velocity of 500 $\mu m/s$). In Fig. S6, we show the trajectories of particles with radii of 110 nm, 90 nm, and 70 nm in the force field, respectively. They are corresponding to the particle sorting in Fig. 6 of the main text. It can be seen that the behaviors of particles of different sizes in the force field are different. It’s the transverse optical force that help to sort these particles at the micrometer scale.


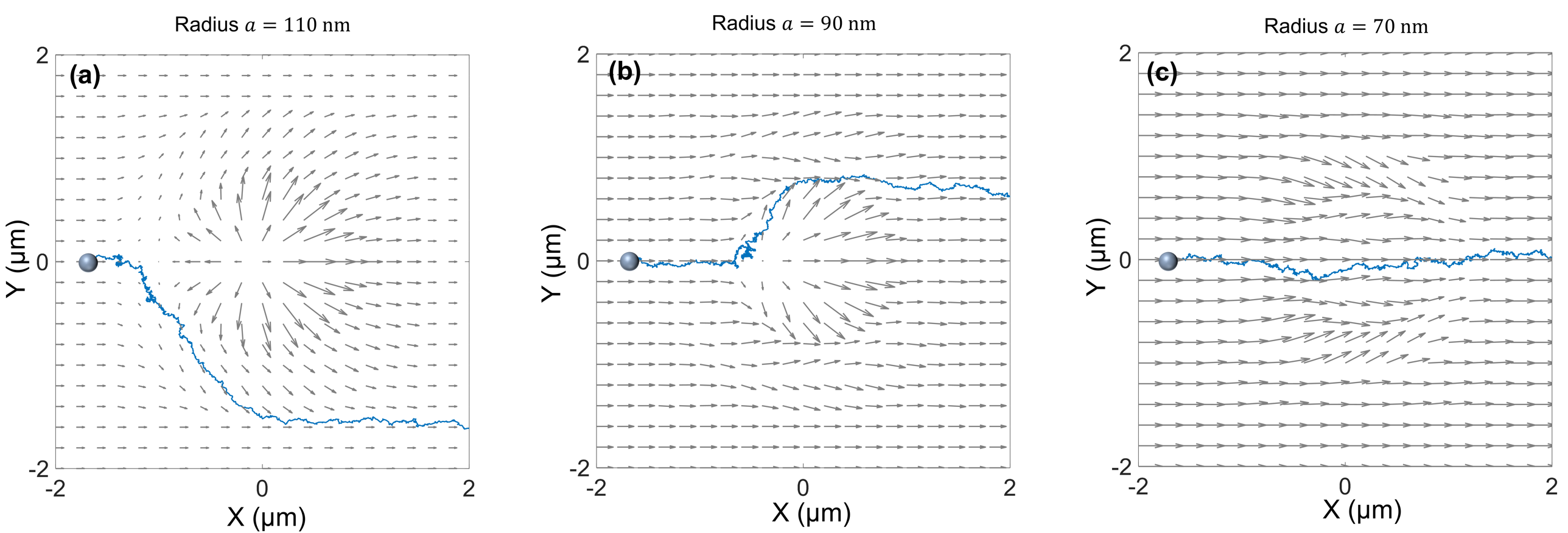


Fig. S6. Simulated force field and trajectory of a gold particle with different radii in the $xy$-plane at $z=2.2 \mu m$. The radii of the gold particles in (a)(b)(c) are 110 nm, 90 nm and 70 nm, respectively. They have been released at the input point (-1.7, 0) $\mu m$ into water flow with current velocity of $500 \mu m/s$. Other parameters are the same as those in Fig. 6 of the main text.

1. Separation/sorting particles with different materials

The sorting system in the main text also enables the separation of particles with different materials. As shown in Fig. S7(a), gold particles and silica particles, which both have a radius of 90 nm, undergo different trajectories. To better illustrate the sorting effect, we plotted the outlet distribution of nanoparticles of both materials in Fig. S7(b).





Fig. S7. Simulation of particle sorting with different materials. (a) Simulated trajectories of gold and silica particles, both with a radius $a=90 nm$ in the $xy$-plane when $z=2.2 \mu m$. (b) Outlet distributions ($x=1.5 \mu m$) of particles with different materials. Other parameters are the same as in Fig. 6 of the main text.

1. 3D optical trapping using an additional counter-propagating beam

In the discussion of off-axis trapping in the main text, we have investigated the force and trapping potential in the transverse plane. For the longitudinal direction, we can use an additional counter-propagating beam to achieve confinement. In Fig. S8(a), the beam waist of the forward-propagating Gaussian beam along the $z$-axis is located at $z=0 \mu m$, and the beam waist of the backward-propagating Gaussian beam is located at $z=4.4 \mu m$. So $F_{z}=0$ at $z=2.2 \mu m$ and there is a potential-well as shown in Fig. S8(b). For the potential calculation, we used $U_{z}\left( z \right)=-\int_{z_{0}}^{z} F_{z}(z)dz$ where $z_{0}=1.5 \mu m$. This means that we have chosen the reference point of energy at $z=1.5 \mu m$ for all y values. In addition, in Fig. S8(c) and Fig. S8(d), the distribution of the transverse force $F_{y}$ and the potential-well $U_{y}$ on the $y$-axis in the $yz$-plane have been shown. We can see that particles can be trapped in 3D at the off-axis location.


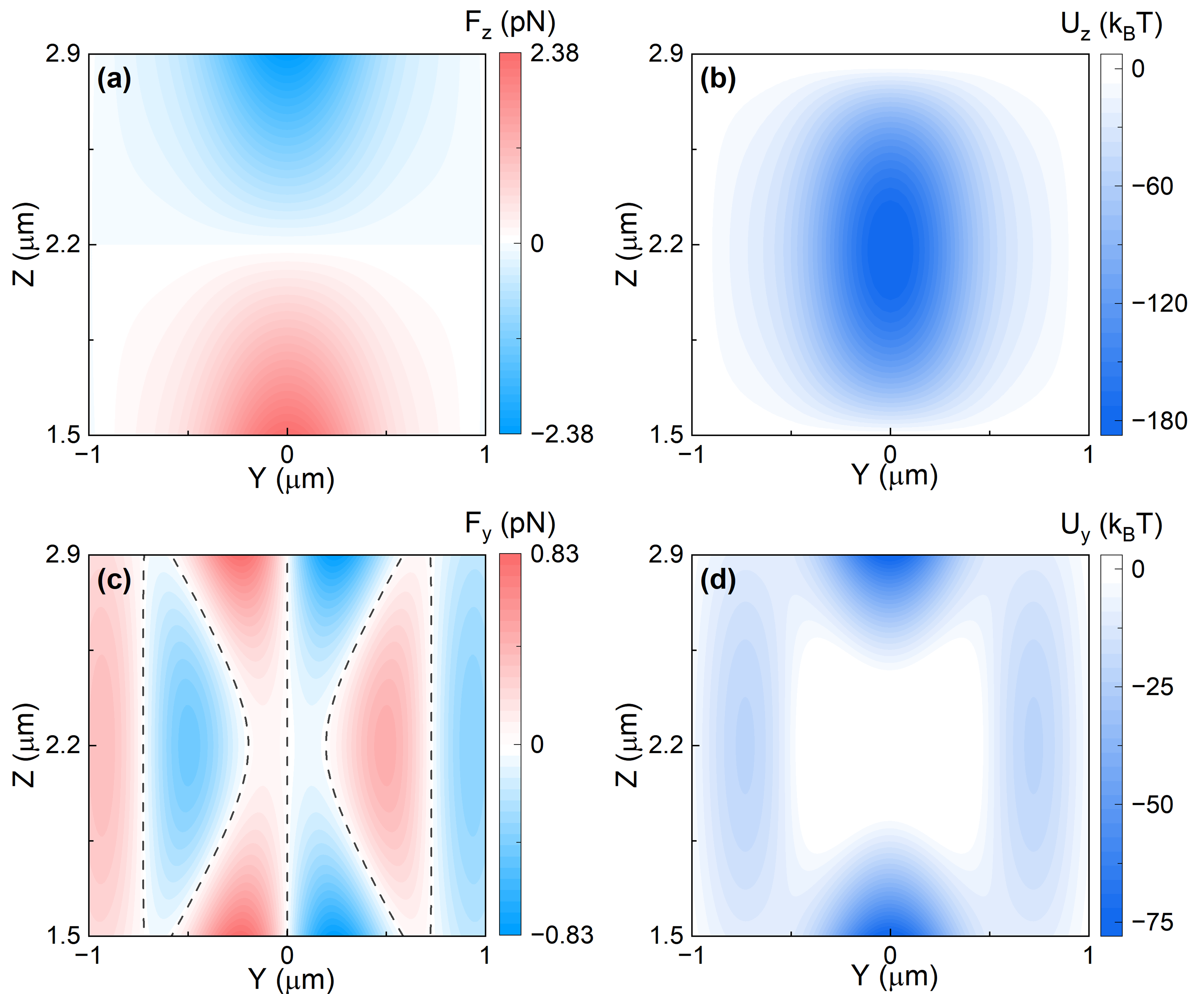


Fig. S8. Numerically calculated forces and potential-wells of a gold nanoparticle with radius $a=80 \mathrm{nm}$ for off-axis trapping in counter-propagating Gaussian beams. (a) Force $F_{z}$ on the $yz$-plane. (b) Potentials $U_{z}$ with unit of $k_{B}T$ ($T=25^{\circ}C$) on the $yz$-plane within the range of $1.5 \mu m<z<2.9 \mu m$. (c) Force $F_{y}$ on the $yz$-plane. The black dashed curves denote the $F_{y}=0$ positions. (d) Potentials $U_{y}$ with unit of $k_{B}T$ ($T=25^{\circ}C$) on the $yz$-plane within the range of $1.5 \mu m<z<2.9 \mu m$. The two laser beams are uncoherent and the power of each beam is $P=50 \mathrm{mW}$. Each beam is linearly polarized in the $x$-direction with a vacuum wavelength $\lambda_{0}=1064$ nm, with the forward-propagating beam waist in the $z=0 \mu m$ plane and the backward-propagating beam waist in the $z=4.4 \mu m$ plane; Other parameters are the same as in Fig. S1.

The trapping ability along the longitudinal direction has been further investigated. Based on the position of the black dashed line in Fig. S8(c), it is known that the off-axis trapping position on $z=2.2 \mu m$ plane is at $y=0.725 \mu m$. The axial forces $F_{z}$ and potential depth $U_{z}$ have been calculated along the $z$ -direction across the point (0, 0.725, 2.2) $\mu m$, as shown in Fig. S9. The potential minimum is at the position of $z=2.2 \mu m$ as designed. We have chosen the reference point of potential energy at $z=1.5 \mu m$. It can be seen that with a counter-propagating beam, the potential depth is about 45 $k_{B}T$ when each beam has a power of 100 mW. This means that particles in the z-direction are well confined in a desired range. As can be seen in Fig. S9(b), most particles are trapped in a range of about 100nm in the z-direction with confined Brownian motion.





Fig. S9. Numerically calculated forces and potential-wells of a gold nanoparticle with radius $a=80 \mathrm{nm}$.for off-axis trapping in counter-propagating Gaussian beams. (a) Force $F_{z}$ along $y=0.725 \mu m$ on the $yz$-plane. $y=0.725 \mu m$ is the off-axis trapping position on the $xy$-plane of $z=2.2 \mu m$. (b) Potentials $U_{z}$ with unit of $k_{B}T$ ($T=25^{\circ}C$) along $y=0.725 \mu m$ on the $yz$-plane within the range of $1.5 \mu m<z<2.9 \mu m$. The laser power of each beam is $P=100 \mathrm{mW}$. Each beam is linearly polarized in the x-direction with a vacuum wavelength $\lambda_{0}=1064$ nm, with the forward-propagating beam waist in the $z=0 \mu m$ plane and the backward-propagating beam waist in the $z=4.4 \mu m$ plane; Other parameters are the same as in Fig. S1.

**References**

[1] L. M. Zhou, K. W. Xiao, J. Chen, and N. Zhao, "Optical levitation of nanodiamonds by doughnut beams in vacuum,", *Laser Photon. Rev.,* vol. 11, no. 2, p. 1600284, 2017.
